# Roles of Transcription Factors in the Development and Reprogramming of the Dopaminergic Neurons

**DOI:** 10.3390/ijms23020845

**Published:** 2022-01-13

**Authors:** Lulu Tian, Murad Al-Nusaif, Xi Chen, Song Li, Weidong Le

**Affiliations:** 1 Center for Clinical Research on Neurological Diseases, First Affiliated Hospital, Dalian Medical University, Dalian 116021, China; tll1205@163.com (L.T.); alnusaif2016@gmail.com (M.A.-N.); lisong@dmu.edu.cn (S.L.); 2 Liaoning Provincial Key Laboratories for Research on the Pathogenic Mechanisms of Neurological Diseases, First Affiliated Hospital, Dalian Medical University, Dalian 116021, China; 3 Institutes of Neurology, Sichuan Academy of Medical Sciences, Sichuan Provincial People’s Hospital, Chengdu 610072, China; cxde2018@163.com

**Keywords:** meso-diencephalic dopaminergic neurons, dopamine, Parkinson’s disease, transcription factors, development, cell therapy, reprogramming

## Abstract

The meso-diencephalic dopaminergic (mdDA) neurons regulate various critical processes in the mammalian nervous system, including voluntary movement and a wide range of behaviors such as mood, reward, addiction, and stress. mdDA neuronal loss is linked with one of the most prominent human movement neurological disorders, Parkinson’s disease (PD). How these cells die and regenerate are two of the most hotly debated PD research topics. As for the latter, it has been long known that a series of transcription factors (TFs) involves the development of mdDA neurons, specifying cell types and controlling developmental patterns. In vitro and in vivo, TFs regulate the expression of tyrosine hydroxylase, a dopamine transporter, vesicular monoamine transporter 2, and L-aromatic amino acid decarboxylase, all of which are critical for dopamine synthesis and transport in dopaminergic neurons (DA neurons). In this review, we encapsulate the molecular mechanism of TFs underlying embryonic growth and maturation of mdDA neurons and update achievements on dopaminergic cell therapy dependent on knowledge of TFs in mdDA neuronal development. We believe that a deeper understanding of the extrinsic and intrinsic factors that influence DA neurons’ fate and development in the midbrain could lead to a better strategy for PD cell therapy.

## 1. Introduction

The meso-diencephalic dopaminergic (mdDA) neurons are the primary dopamine (DA) sources in the mammalian central nervous system [1]. DA, as a neurotransmitter, plays a critical role in responding to ever changing environmental conditions, such as movement, reward, punishment, salience, learning, cognition, love, pleasure, and drug addiction [2,3,4,5,6]. Importantly, mdDA neuronal loss is linked with one of the most prominent human movement neurological disorders, Parkinson’s disease (PD) [7]. PD is the second most common neurodegenerative disease, characterized by motor symptoms including static tremor, rigidity, bradykinesia, postural disorder, and non-motor symptoms including sensory and cognitive impairment [1,8]. The main pathological feature of PD is the irreversible mdDA neuronal degeneration in the substantia nigra pars compacta (SNpc), which primarily impairs striatal dopaminergic innervation and causes cardinal motor symptoms [1,9].

Currently, the main treatments for PD still focus on increasing DA levels or regulating DA transmission by pharmacotherapy. Deep brain stimulation is also applied for patients who experience a prominent tremor or uncontrolled motor fluctuations [10]. However, these treatments can only alleviate the physical symptoms rather than prevent or delay the disease progression [11]. Especially, long-term pharmacotherapy treatments might have serious adverse effects, such as dyskinesia and impulsive control disorders [12]. Cell transplantation, a potential strategy to replace impaired mdDA neurons, has gained particular interest [13]. Previously, brain cells were regarded as non-renewable cells. However, discovering neural stem cells (NSCs) makes brain cell regeneration feasible and brings new insights into incurable neurodegenerative disorders. After decades of research, it was found that stem cell therapy in PD can selectively slow the disease progression, and, more importantly, it may potentially resolve the root problems in the future [14]. Thus, tremendous effort and research have been focused on the vital step for stem cell therapy of generating functional mdDA neurons by cell reprogramming from stem cells or other non-neural cells [15,16,17]. As a result, a serial of transcription factors (TFs) has been characterized, which are essential elements in mdDA neuronal development, differentiation, specification, and transmitter synthesis.

mdDA neuronal development and differentiation mechanisms are highly complex and influenced by multiple genes and factors. TFs are protein molecules that bind to a specific gene sequence and ensure that the target gene is expressed at a particular time and space [18]. They can control the specific cell types and the process of cell development [19]. Aldehyde Dehydrogenase 1 family member A1 (Ascl1), Neurogenin2 (Ngn2), Neuronal differentiation 1 (NeuroD1), Forkhead box A2 (Foxa2), LIM homeobox transcription factor1 A (Lmx1a), Nuclear receptor-related factor1 (Nurr1), and Paired-like homeodomain3 (Pitx3) are among the TFs implicated in mdDA neuronal development and maturation [19,20,21]. Ascl1, Ngn2, and NeuroD1 are neural progenitor cell markers, whereas Pitx3 and Nurr1 are mdDA neuron-specific markers. These TFs have long been known to involve the differentiation, maturation, and maintenance of mdDA [15,16,17]. Additionally, some neurotrophic factors, such as brain-derived neurotrophic factor (BDNF) and glial-cell-derived neurotrophic factor (GDNF), also play an essential role in this process. Unlike performing in isolation, all of these factors interact to a variable degree, i.e., they are not only involved in the early neuronal events but are also continuously expressed during neuronal maturation and even throughout adulthood. In this review, we first seek to encapsulate the mechanism discovered to be underlying mdDA neuronal development via the TFs and focus on the application of this updated knowledge in stem cell therapy, i.e., how to promote the differentiation of stem cells into dopaminergic neurons (DA neurons) in vitro or in vivo and even within the therapeutic target.

## 2. TFs in Development of mdDA Neurons

mdDA neurodevelopment involves a series of events that begin with the midbrain floor plate (FP) induction and progress through the specification of neuron progenitors, differentiation of these progenitors, maturation, migration, and the formation of synaptic neural circuits. Several factors and specialized morphogens guide these actions at each stage of development. This section will go over the principles of mdDA neurodevelopment and elucidate the TFs’ roles in promoting mdDA neurodifferentiation.

### 2.1. Induction of Midbrain FP

**Figure 1:**
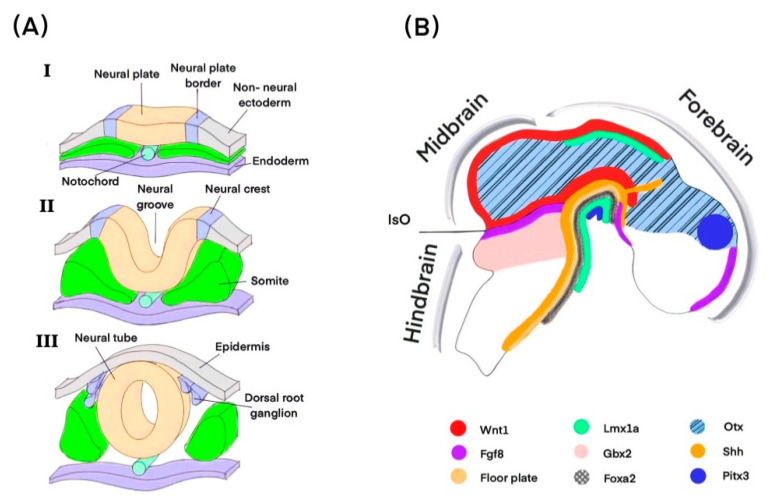
Development of brain cells. (**A**) Neural tube formation (**I**–**III**). (**B**) TFs, morphogens, and signaling involved in mdDA neuron formation. Otx and Gbx2 function in opposition to one another to establish the position of the IsO, which defines the midbrain-hindbrain boundary. IsO regulates Fgf8, which, along with Shh, specifies the location of midbrain mdDA neuron growth. Shh stimulates Foxa2 expression, and Wnt1 is expressed in this region and required for midbrain development. Lmx1a is defined in the ventral midbrain; Pitx3 plays a role in the mdDA neuron differentiation. Abbreviations: TFs, transcription factors; IsO, isthmic organizer; Otx, Orthodenticle homeobox 2; Gbx2, Gastrulation brain homeobox 2; Fgf8, Fibroblast growth factor 8; Foxa2, Forkhead box A2; Lmx1a, homeobox transcription factor 1 A; Pitx3, Paired-like homeodomain3; and Shh, Sonic Hedgehog.

**Figure 2:**
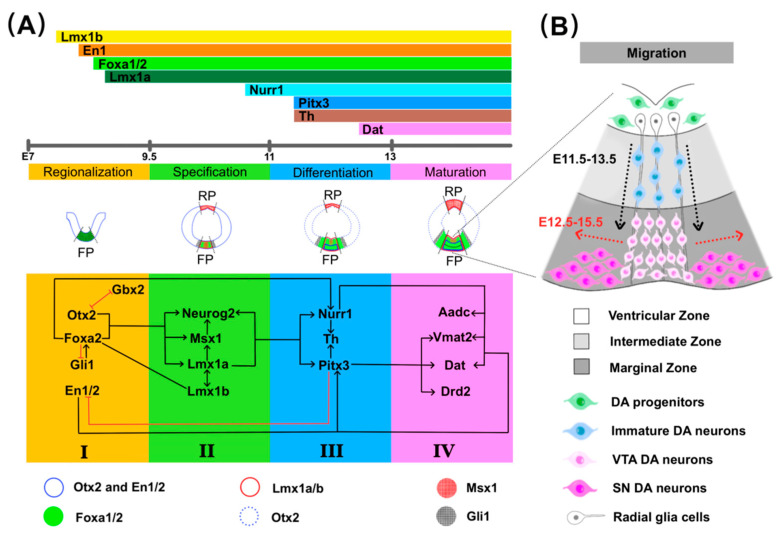
TFs and related molecules involved in developing mdDA neurons at various embryonic stages. (**A**) mdDA neuron induction, specification, differentiation, and maturation. The use of arrows denotes stimulatory effects, while perpendicular lines denote inhibitory effects. (**A**(**I**)) The orange area clusters the TFs and molecules involved in forming mdDA neurons from the regional specification and induction stage (see text for details). (**A**(**II**)) The green section groups together the TFs involved in the FP specification (see text for details). (**A**(**III**)) The TFs and related molecules implicated in mdDA differentiation are depicted in blue. (**A**(**IV**)) The pink area represents the TFs’ involvement in the expression of mdDA neurons maturation markers. (**B**) The mdDA neuron migration paths. Black dotted arrows indicate radial migration regulated by chemokine (C-X-C motif) and ligand 12 (CXCL12). The red dotted arrows indicate tangential migration regulated by the L1 cell adhesion molecule (L1CAM), the L1CAM ligand-protein tyrosine phosphatase (L1CTP), and the reelin signaling pathway. Abbreviations: FP, floor plate; RP, roof plate; Gli1, Glioma-associated oncogene homolog1; En1, Engrailed-1; Msx1, Msh homeobox 1; Nurr1, Nuclear receptor-related factor1; Th, tyrosine hydroxylase; Aadc, amino acid decarboxylase; Vmat2, vesicular monoamine transporter 2; Dat, dopamine transporter; Drd2, dopamine D2 receptor.

When the ectoderm thickens to form the neural plate, the trajectory of neural development begins. This plate begins to fold inward and merge, resulting in neural tube formation (Figure 1A). Recent research has identified that the sex-determining region Y-box 2-to-Brachyury (Sox2-to-Bra) ratio is involved in neural tube specification [22]. The neural tube consists of a cluster of neural progenitor cells (NPCs) and post-mitotic neurons. Foxa2 and its downstream effector RNF152 regulates cell proliferation via the mTOR pathway to influence FP cell number [23]. The FP is a neural tube’s ventral midline signaling center, where mdDA neurons come from, while the roof plate (RP) is placed in the dorsal midline [24]. The ectoderm and the RP produce a bone morphogenic protein (BMP) [25]. Shh is secreted from the notochord underlying the ventral neural tube and the FP [26]. Foxa2 regulates the expression of sonic hedgehog (Shh), which in turn activates Glioma-associated oncogene homolog 1 (Gli1) and Foxa2 (Figure 2A(I)). The dorsoventral (DV) axis is defined by BMP and Shh morphogen gradients. The precise spatial and temporal interplay of signaling along the DV axis was used to specify NPCs into different neuronal types in each designated area [27,28].

As the neural tube develops, it generates four major morphogenetic domains along the anterior–posterior (A–P) axis: the prosencephalon, the mesencephalon, the rhombencephalon, and the end of the neural tube, which will eventually form the cerebrum, the midbrain, the rest of the brainstem, the cerebellum, and the spinal cord, respectively (Figure 1B). The isthmic organizer (IsO), which defines the midbrain–hindbrain boundary (MHB), appears during neural tube development on embryonic days 7–8 (E7–8). The IsO function is similar to the medial FP in that it is an important signaling center for mdDA neuron formation, and it determines the separate embryonic development of the midbrain and hindbrain [29,30]. The position of IsO is determined by the expression boundaries of TFs Orthodenticle homeobox 2 and Gastrulation brain homeobox 2 (Otx2 and Gbx2) in the central nervous system: the former is expressed in the forebrain and midbrain. At the same time, the latter is defined in the anterior hindbrain. Disruption of the Otx2/Gbx2 border causes an IsO positional shift with an expanded or diminished mid or hindbrain. Fibroblast growth factor 8 (Fgf8) is solely expressed on the hindbrain side of IsO, whereas Wingless-type MMTV integration site family member 1 (Wnt1) is expressed on the midbrain side (Figure 1B) [31,32]. The primary role of Wnt1 is to regulate mdDA neuron development and maintain the expression of Engrailed-1 (En1), which may act through increasing En1 transcriptional activity to induce midbrain specification [33,34]. Furthermore, TFs including En1/2, Foxa1/2, Gbx2, Otx2, and secreted factors including Wnt1, Fgf8, and Shh are fundamental for induction and specification mdDA progenitor cells (Figure 1B) [24,35,36,37]. However, the interaction between morphogens and TFs is still not fully understood, which is crucial in exploring these mechanisms further.

### 2.2. Specification of mdDA Progenitors

The programming of FP cells results in the induction of the NPCs into mdDA progenitors, which is essential for the specification of mdDA progenitors. It has been demonstrated that a significant number of TFs are involved in this process. The first expression of Lmx1a appears around E8.5 in the midbrain FP, and the FP cells are differentiated into mdDA progenitors by expressing Foxa2 and Lmx1a [38]. Lmx1a/b cooperate to regulate neurogenesis via Msh homeobox 1 (Msx1)-mediated Ngn2 regulation (Figure 2A(II)) [24,36]. Furthermore, Lmx1a/b plays an essential role in regulating the proliferation of mdDA progenitors by Wnt1 and Ngn2 [39]. The number of mdDA progenitors and mdDA neurons reduces significantly in Lmx1a knock-out and dreher mice, partly attributable to a loss in neurogenesis and proliferation of mdDA progenitors [39,40]. However, the specification of mdDA progenitors failed to be affected in specific inactivation of Lmx1b in FP at E9. Therefore, Lmx1b can act as a compensatory function for Lmx1a by promoting the specification and proliferation of mdDA progenitors.

The study has shown that the absence of En1 does not affect the identity of mdDA progenitors and early post-mitotic neurons, but the down-regulation of some mdDA neuron markers (Pitx3; tyrosine hydroxylase, Th; dopamine transporter, Dat; vesicular monoamine transporter 2, Vmat2; and amino acid decarboxylase**,** Aadc) was found in the rostral–lateral DA domain of En1^−/−^ embryos [41]. Therefore, En1 may play a more critical role in the maturation of mdDA neurons than specification. Foxa1/2 have many overlapped bound sites, suggesting their cooperating role in developing mdDA neurons. Foxa1/2 activates Lmx1a/b, which regulates the specification and differentiation of mdDA neurons [39,42]. Moreover, Foxa1/2, an E-box transcription factor and Otx2 are directly involved in the transcriptional activity of a Neurog2 enhancer during the differentiation of mdDA neurons. Neurog2 starts to express at E10.75 in the rostral FP later than all three TFs, suggesting Neurog2 needs to be activated by other factors [43]. Otx2 plays an essential role in positioning MHB and regulates progenitor domains’ specification in the ventral midbrain (VM). It has been identified that Otx2 conditional deletion in the VM results in abnormal expression of Shh, NK homeobox factor 6.1 and NK homeobox factor 2.2 (Nkx6.1 and Nkx2.2) and changed mdDA progenitors specification [44]. Furthermore, the Otx2 deletion in VM impairs the proliferation of Sox2^+^ mdDA progenitors, resulting in the inhibition of Lmx1a, Msx1, Ngn2, and Ascl1 expression in mdDA progenitors. Otx2 overexpression increases the number of mdDA neurons but not those in other progenitor domains, implying that Otx2 can selectively regulate the proliferation of mdDA progenitors [45].

### 2.3. Differentiation and Maturation

After completing the cell cycle and entering the post-mitotic stage, mdDA progenitors differentiate into mature mdDA neurons. Nurr1 continues to be expressed in the midbrain from E10.5 to adulthood [46]. Nurr1 can influence mature mdDA neuron markers such as Th, Dat, Aadc, and Vmat2 (Figure 2A(III)), and Nurr1-deficient mice cannot develop mdDA neurons and die shortly after birth [46,47]. In either Foxa1 or Foxa2 conditional deletion at E11.5, the number of Nurr1^+^ post-mitotic mdDA neurons is reduced, and the differentiation from Nurr1^+^ immature to Nurr1^+^ Th^+^ mdDA neurons is inhibited. Foxa2 has been found to influence neuronal differentiation by binding directly to a Neurog2 enhancer and activating Smarca1, promoting the induction of mature mdDA neurons in the developing mouse midbrain [43]. Furthermore, Foxa2 binding to Nurr1 promotes the expression of DA phenotypes throughout the development of mdDA neurons [48,49].

The onset of Th and Pitx3 expression is approximately around E11.5 [50]. The ventrolateral mdDA neurons express Pitx3 before Th, whereas the dorsomedial ones express Th before Pitx3. In the absence of Pitx3, SNpc neurons lose at the beginning of their terminal differentiation. Consistent with this, a recent study demonstrates that the lack of Pitx3 in *Pitx3^fl^*^/*fl/*^*Dat^CreERT2^* mice causes a rapid reduction of DA content and a severe loss of mdDA neurons but not ventral tegmental area (VTA) [51]. Therefore, Pitx3 is essential in the terminal differentiation of the SNpc [52]. Other TFs, including En1/2 and Otx2, also play crucial roles in the differentiation and maturation of mdDA neurons. Previous studies have indicated that En1-null mice have a significant loss in the entire SNpc and most VTA [41,53]. En1/2 is also required to survive the mature mdDA neurons (Figure 2A(III,IV)) [54,55]. Overexpressed Otx2 in the midbrain results in the increase of Nurr1^+^ immature mdDA neurons and Pitx3^+^ mature neurons through enhancing the proliferation of mdDA progenitors, while the deletion of Otx2 leads to the failure of mdDA progenitors differentiation into Nurr1^+^ post-mitotic mdDA neurons because the expressions of Lmx1a, Msx1, Ngn2 and Mash1 in mdDA progenitors are lacking. Therefore, Otx2 might regulate the expression of Lmx1a to involve mdDA neurons’ differentiation.

### 2.4. Migration

The differentiation and maturation of mdDA neurons are accompanied by their migration. mdDA neurons migrate away from the FP of the ventral mesencephalon, called the ventricular zone (VZ), into the mantle layer and form three different mdDA neuron clusters: SNpc (A9) on the lateral side, VTA (A10) on the medial side, and the posterior retro-rubral field (RRF; A8) [37]. Previous research shows that mdDA neurons migrate first radially between E11.5–13.5 after exiting the cell cycle, and then some mdDA neurons begin to migrate tangentially into the laterally-situated SNpc medially-located VTA at E12.5 (Figure 2B) [56]. Furthermore, not all SNpc mdDA neurons migrate tangentially after radial migration; some mdDA neurons migrate radially into the dorsal SNpc [57]. It has been proposed that the chemokine (C-X-C motif) ligand 12 (CXCL12) and its receptor CXCR4 [58] are involved in regulating the radial migration, and L1 cell adhesion molecule (L1CAM) and potential L1CAM ligand–protein tyrosine phosphatase, receptor type Z, polypeptide 1 (PTPRZ1) [37] modulate the tangential migration of mdDA neurons as well as Reelin signaling [56,59]. Recent research found that Netrin-1 mediates dorsal mdDA neuron migration into SNpc along radial glia fibers, and axon-derived Netrin-1 attracts GABAergic neurons into substantia nigra pars reticula (SNr) [57]. Moreover, early B-cell factor 1 (Ebf1), an essential TF in B-lymphocyte differentiation, has been implicated in mdDA neuron migration. Cellular Ngn2 disruption causes both an arrest of cell migration and a failure of cell differentiation [60].

During the differentiation of mdDA progenitors at E11.5, Sox2^+^ Lmx1a^+^ progenitors with low Sox6 expression are located in the medial VZ domain, while Nolz1 expresses laterally with high Sox6 expression. After exiting the cell cycle, mdDA neurons begin to migrate: Sox6^+^ mdDA neurons, located in the medial area, migrate ventrally and then tangentially to end up in the lateral SNc at E18.5; Otx2^+^ or Otx2^+^ Nolz1^+^ cells, positioned in the lateral domain, extend ventrally and are confined to the VTA [61]. Furthermore, many studies have found that Otx2 is limited to VTA neurons at the post-mitotic stage [62,63]. Even with what has already been done, it is still difficult to ascertain how Otx2 and Sox6 are involved in mdDA neuron migration and the precise interaction mechanism between these TFs and mdDA neuron subtypes.

## 3. TFs in Dopamine Cell Therapy

### 3.1. Dopamine Cell Therapy

The current pharmacological approach for PD patients is still the primary management to alleviate or control motor symptoms. The treatment is generally aimed at increasing DA bioavailability, either by replenishing the DA precursors or inhibiting DA’s breakdown. However, it cannot directly replace the lost pathway. Regenerative medicine-based solutions are being aggressively pursued to restore dopamine levels in the striatum via several emerging techniques designed to reconstruct the nigrostriatal pathway. Currently, several different types of stem cells have attempted to regenerate mdDA neurons. Due to their self-renewing and multipotent features, the most commonly used stem cells are embryonic stem cells (ESCs), mesenchymal stem cells (MSCs), and pluripotent stem cells [24]. Several trials with grafts integrated into the host brain have shown restored DA release, re-innervated striatum, and alleviated clinical symptoms of motor dysfunction. In certain hopeful situations, patients may be able to discontinue L-dopa medication following transplantation [64,65]. However, multiple concerns remain that most in vivo transplanted NSCs become glial cells rather than neurons, and only 5–10% of NSCs survive after transplantation due to the toxic effect of the inflammatory state [66,67,68]. As a result, it is critical to identify a method to protect DA neurons from neuroinflammation and enhance their survival and differentiation. Among the most effective and attractive methods, TFs-based therapeutics have gained considerable interest. The TFs involved in enhancing mdDA neuron cell development will be reviewed in depth below, and they can be employed alone or in combination.

### 3.2. Nurr1

Nurr1, also known as NR4A2/NOT/TINUR, is a member of the orphan nuclear receptor family 4 (NR4A), necessary for the mdDA neurons’ development, maturation, and functional maintenance [46,69,70]. It is also involved in neuroprotection and neuroinflammation regulation by inhibiting pro-inflammatory factors in microglial and astrocytes cells [71,72,73]. Notably, in mdDA neurons, the decreased levels of Nurr1 were characterized during aging, which may be related to the increased morbidity of PD [74,75]. Furthermore, Nurr1 is required for neuronal plasticity remodeling. Previous research found two functional Nurr1 binding sites in the proximal Topoisomerase IIβ (Top IIβ) promoter [76], since it was known that Topo IIβ deficiency affects axon growth through Rho-GTPase dysregulation [77]. Additionally, genome-wide analysis in human NSCs identified many Nurr1 direct genes involved in synapse formation [78]. However, little is yet known about how Nurr1 regulates synaptogenesis. Moreover, Nurr1 appears to be an essential TF for maintaining mdDA neurons’ distinct traits by positively regulating many nuclear-encoded mitochondrial genes [79], manifested by the evidence that mitochondrial impairment modulated by MPP (+) was improved by Nurr1 agonists’ treatment [80].

Nurr1 is required for DA phenotype genes (Th, Dat, and Vmat2) expression. Overexpression of Nurr1 was thought to induce the development of mature DA neurons during cell differentiation. ESCs and embryo-derived cells were the most common cells used in cell reprogramming due to their pluripotency. As expected, Nurr1 transduction promotes in vitro differentiation of mouse ESCs into mature DA neurons (Table 1) [81,82]. Co-expressing Nurr1 and GPX-1 (Glutathione peroxidase 1, a neuroprotective enzyme against oxidative stress) in mouse ESCs results in the differentiation of DA-like cells with increased survival ability [83]. Additionally, exogenous Nurr1 expression in embryonic corticoid-derived NPCs and embryonic midbrain-derived NSCs achieves comparable results to the above ESCs [84,85]. In the presence of Nurr1-Mash1, thyroid hormone derivatives have been shown to stimulate differentiation of embryonic corticoid-derived NPCs into DA neurons [84]. Besides this, these derivatives have been displayed in vitro to protect DA neurons from neurotoxic damage caused by 6-hydroxydopamine (6-OHDA) and hydrogen peroxide (H_2_O_2_). Similarly, Urocortin (UCN), a corticotropin-releasing hormone family peptide, has been shown to enhance Nurr1^+^ NPCs differentiation into Th^+^ DA neurons in vitro and in vivo as Nurr1, Foxa2, and Pitx3 expression [85].

Previous studies have found that most transplanted NSCs fail to differentiate into neurons but glial cells and survive after transplantation [66], which might significantly contribute to a poor host cellular environment. In light of the role of Nurr1 in glial cells [40,72], co-culture of embryonic mesenchymal NSCs and primary microglial cells overexpressing Nurr1 prolonged the survival of transplanted NSCs, decreased the number of microglial cells and showed long-term survival [86,87]. Recent innovative research has found that co-grafting NNSC and NMG (NSCs and microglia both with Nurr1 overexpression) improved the behavior of PD rats. Furthermore, with a better understanding of MSCs’ accessibility, multilineage potential, and non-tumorigenic potential, MSCs are thought to have a better potential for clinical applications without ethical constraints [88]. A recent study reveals that the grafts of MSCs overexpressing Nurr1 in the striatum of PD rats not only survive and migrate in the brain but also alleviate PD symptoms, increasing the number of Th^+^ cells in the SNpc, and, most importantly, inhibiting glial cell activation [89].

### 3.3. Pitx3

Pitx3 is a TF required for the development and survival of mdDA neurons [110]. After birth, Pitx3 is expressed constitutively in the midbrain’s SNpc and VTA. ESCs are also used to study Pitx3’s role in PD cell therapy. Research shows that exogenous Pitx3 in ESCs-derived progenitor cells promotes the generation of DA neurons in vitro through regulating the expression of Th, Ngn2, and β-tubulin Ⅲ genes [91]. High Th expression was also significantly correlated with increased Pitx3 expression [111]. In vivo, ESCs have been shown to have the potential for PD cell therapy [13]. Correspondingly, transplanting ESCs-derived progenitor cells overexpressing Pitx3 restores the functional deficits in PD rats. However, grafts of Pitx3-eGFP^+^ or Pitx3-eGFP^−^ cells sorted from these cells show a different result: cell grafts from Pitx3-eGFP^−^ cells improve motor behavior deficits, but not Pitx3-eGFP^+^ cells [92]. Furthermore, the size of the Pitx3-eGFP^+^ cell grafts decreases [92], implying that these grafts do not survive and integrate into the host brain. The timing of Pitx3 expression could cause this during DA neuron development. The onset of Pitx3 expression coincides with the cell cycle exit and the entry into the post-mitotic state. Thus, a poor survival rate may be achieved following transplantation. On the other hand, incorporating GDNF and GFR-1 into embryonic brain-derived NSCs could improve the behavior of PD models. In a rat PD model, GDNF, and its receptor GFR1 signaling, activate Nurr1 and Pitx3 to increase the survival of transplanted midbrain-derived NSCs [112,113]. During embryogenesis, Pitx3 is required to activate BDNF expression in a rostro-lateral SNpc mdDA, and GDNF transient expression in the murine VM induces Pitx3 transcription via NF-B signaling. Meanwhile, overexpression of Pitx3 protects mdDA neurons by increasing GDNF and BDNF expression [114,115]. Thus, more research may be needed in the future to determine whether co-transplantation of non-neuronal cells overexpressing Pitx3 with NSCs promotes graft survival and integration.

### 3.4. Lmx1a/b

Lmx homeodomain TFs (Lmx1a/b) are required to develop mdDA neurons. Lmx1a is expressed first at E8.5 in the RP [116]. During mdDA neurogenesis, Lmx1a is expressed by the mdDA progenitors and maintains its expression in the post-mitotic mdDA neurons. Lack of Lmx1a/b results in impaired respiratory chain activity, increased oxidative stress, mitochondrial DNA damage, and axonal pathology. These disturbed molecular pathways eventually lead to synuclein accumulation and autophagy defects, as well as the loss of mdDA neurons [117]. Moreover, it has been known that Lmx1a/b are essential for mdDA neuron excitatory synaptic inputs and dendritic development [118]. As previously stated, Lmx1a is more important in developing mdDA, and Lmx1b acts as a compensatory factor for Lmx1a. It was reported that Lmx1a, Foxa2 and Pitx3 could increase Th expression when delivered to NPCs during neural proliferation, but only Lmx1a increases expression after induction, which is more efficacious [15]. When embryoid body cells (EBCs) transplanted into the adult intact SNpc, they differentiate into NPCs without acquiring DA phenotypes. In contrast, EBCs that overexpress Lmx1a develop DA neuronal markers. Notably, when EBCs are transplanted into 6-OHDA-lesioned SNpc, they develop into Th+ cells without the need for exogenous Lmx1a expression. However, these Th+ cells did not survive long [94], implying that additional factors are required to ensure long-term survival and terminal differentiation. Furthermore, Lmx1a-eGFP^+^ cell grafts are more predictable and enriched in DA neurons when isolated from ESC-derived progenitor cells overexpressing Lmx1a and transplanted into PD rodents. This study demonstrated that the appropriate integration might accelerate functional recovery. Additionally, mature Th^+^ DA neurons from Lmx1a-eGFP^+^ cell grafts are two-fold higher than unsorted grafts [92], indicating that pure grafts are more predictable and safer, which may be a future trend in PD cell therapy.

### 3.5. En1

En1 expression starts in the mid and hindbrain at E8 [119]. En1 protein-coding genes are linked to NSCs and lineage-specific markers [24,120]. Paradoxically, in the absence of En1, the ectopic expression of mdDA neuron markers was diminished in the metencephalon, suggesting that En1 influences the relocation of MHB [121]. It is proposed that En1 is significant for the correct establishment of IsO by controlling the proper expression of Fgf8, Otx2, and Wnt1 [122]. Overall, En1 is essential for the appropriate maintenance and function of IsO [20,122]. In addition, En1 induces epigenetic modifications in the nucleus, involves the guidance of retinal ganglion cell axons and maintains synapse integrity by influencing mitochondrial function [123]. Similarly, En1 can also protect mdDA neurons from oxidative stress and preserve axonal integrity [124,125]. The combination of En1 and other TFs has been certified to reprogram cells into DA neurons successfully [17,108] (Table 1).

### 3.6. Foxa1/2

Despite being required for Lmx1a/b, Nurr1, En1, Aadc, and Th expression, Foxa1/2 are critical TFs in the early development, specification, and maturation of mdDA neurons [42,126]. Foxa1/2 are also involved in neuron projection development and axon guidance [43]. Aside from that, Foxa2 is necessary for Foxa1 expression at E8.5, and Foxa1 has been shown to induce the differentiation of pluripotent P19 cells into neural stem-like cells [127]. Recent studies found that Foxa1/2 deletions resulted in down-regulation in Th and DA synthesis, as well as burst-firing activity in SNpc mdDA neurons [128,129]. As previously stated, Foxa1/2 plays overlapping roles in the specification and development of mdDA neurons. Furthermore, Foxa1/2 also regulates Shh expression. While Foxa2 deficiency causes a transient decrease of Shh, Foxa1 deficit does not. Foxa1 and Foxa2 both positively and negatively control Shh signaling to determine the identity of ventral midbrain progenitors [130]. Therefore, more research now focuses on the generation of DA neurons mediated by Foxa2 rather than Foxa1. As stated below and in Table 1, Foxa2, like other TFs, has been identified as a potentially excellent forward-looking DA inducing neuron when combined with other TFs.

### 3.7. Combinations of TFs

As noted previously, transplanted NSCs primarily differentiated into glial cells rather than DA neurons in vivo, resulting in a poor outcome. As a result, it is necessary to develop a method to protect DA neurons from neuroinflammation and promote the survival and differentiation of transplanted NSCs into DA neurons [87]. Forced expression of ALN (Ascl1, Nurr1, and Lmx1a) in glial cells resulted in the generation of DA neurons with DA release and spontaneous pace-making activity [102,104]. In contrast, neurons derived from astrocytes or NG2 glial do not express Th in vivo [131,132]. Transplantation of human astrocytes reprogrammed by NeAL218 (Neurod1, Ascl1, Lmx1a, and microRNA miR218) can generate induced DA neurons (iDANs) directly in vivo, which are capable of expressing typical DA neuron markers, adopting mature neuronal morphology and functionality, and rescuing some of the motor behavior in PD animal models. Unlike the above two types of research, these iDANs do not derive from or acquire a striatal neuron phenotype [16]. Even so, reprogramming efficiency alone is insufficient, and the next step is to improve its efficiency and safety in humans. A new strategy for co-grafting NPCs and midbrain-derived astrocytes with Nurr1 and Foxa2 overexpression has recently been revealed, boosting graft maturation and survival and resulting in an improvement in the therapeutic impact on NPCs cell transplantation. [97]. Astrocytes are transformed from pro-inflammatory, immunogenic astrocytes to regeneration-friendly astrocytes when activated by region-specific TFs [133]. Only these neurotrophic, anti-inflammatory astrocytes promote the maturation and survival of VM-NPC transplants.

Although ESCs or embryo-derived cells are being studied for PD cell therapy, many technical and ethical issues remain. Induced pluripotent stem cells (iPSCs), derived from adult somatic tissues and differentiated into specific cells similar to ESCs, remove ethical barriers and reduce the likelihood of immune rejection. A growing body of research has explored how to make self-renewing NPCs from fibroblasts instead [95,101,134]. In vitro, mouse embryonic and postnatal fibroblasts can be transdifferentiated into induced neural progenitor cells (iNPCs) using the Wernig factors Ascl1, Brn2, and Myt1 [95,101], but their regional identity is lost, and iNPCs preferentially differentiate into astrocytes. Likewise, mature DA neurons were generated from iNPCs forced expression of Nurr1 and Foxa2, but the neuronal maturity of engrafted neurons was different between in vivo and in vitro when the iNPCs were transplanted into the striatum of PD rats [95], which may result from poor graft quality, immune response or poor host microenvironment. Recent studies found that autologous iPSCs derived from human skin fibroblasts are obtained through a new reprogramming method combining TFs (Oct4, Sox2, Klf4, and c-Myc) and miRNAs (miR-302s, and miR-200c) [109,135]. The transplantation of differentiated and purified iPSCs-derived NPCs into PD animal models can restore motor symptoms and form functional connections with other neurons without tumorigenicity or toxicity [109,136,137], implying that purified graft cells are safer and more predictable. More notably, cells have been transplanted into PD patients in a clinical trial [138] and a case report indicates that a patient with PD may benefit from these autologous NPCs [135]. Autologous transplantation can avoid host immune rejection. Nevertheless, graft quality, including mutations and contamination, is crucial because it is linked to the safety of transplantation trials [139]. Additionally, the source of the iPSCs is a concern: it is unknown if autologous iPSC-derived NPCs from PD patients can develop defective DA neurons. Even when iPSC-derived DA neurons from genetically related healthy donors are transplanted, the host brain’s inadequate microenvironment may affect healthy grafts [13]. The results of ongoing clinical trials will provide us with a clear idea regarding keeping this strategy safe and efficient for a long time.

Moreover, some studies directly converse fibroblasts into DA neurons by different TF combinations [100,107,108]. The combination of six TFs (Ascl1/Nurr1/Lmx1a/Pitx3/En1/Foxa2) could induce the expression of DA neuronal marker genes more efficiently than any other combination. Importantly, when combined with Shh and Fgf8, these six factors can induce mature DA neurons in vitro [108]. In addition, micro-34b/c has been shown to promote cell cycle exit by regulating Wnt1 and enhancing mesencephalic DA neuron differentiation when combined with Ascl1 and Nurr1 [100]. Therefore, small chemical molecules also influence neuron differentiation as well as TFs [36]. However, the grafts subsequently integrate and survive poorly in host tissues after transplantation and further research is needed to improve these issues. Multiple methods using TFs combinations to induce DA neurons are shown in Table 1.

Cell reprogramming provides an ideal strategy for generating DA neurons from non-neural cells. However, induced DA cells by simple overexpression in the developing midbrain are prone to immature neurons and short maintenance for their phenotype after transplantation [140,141]. It is important to note that tumors may form when grafting undifferentiated cells or immature neural progenitors into the therapy. Future research may focus on using both forced expression of compound TFs and co-grafting different cell types simultaneously to promote DA neuron generation at specific regions and times and to help the graft mature and survive. iPSCs still have a promising future in PD cell therapy. Moreover, many unanswered questions still need to be further addressed [95,142]: to what extent, if any, do the various TFs described above interact? what are their downstream targets and upstream activators? to obtain the best results, which sources of cells need to be utilized (xeno or allotransplantation; cell types; pure DA neurons or other cells)? and how to prevent cancer formation?

## 4. Conclusions

The evolution of the DA system has been a topic of extensive research in recent years. Many molecules have been demonstrated to facilitate mdDA neuron development. Studies of TFs that play critical roles in various stages have greatly improved our understanding of how these neurons are generated and paved the way for developing new strategies for transplantation therapy. In vitro and in vivo, using isolated or mixed TFs has successfully induced the generation of DA neurons, which manipulate cell fate for a specific cell type. The grafts from neural cells induced by TFs can survive and play a DA neuron’s role in PD model animals, such as DA release and motor behavior improvement. However, many critical issues need to be further explored regarding DA neurons’ differentiation, survival, and maturation after iNPCs transplantation. The clinical trials are the final manifestation to determine the efficacy of cell transplant therapy. We believe that in-depth knowledge of the critical regulatory proteins and hierarchical networks involved in mammalian mdDA neuron differentiation will benefit future clinical applications and regenerative medicine.

## Figures and Tables

**Table 1:** Generation of DA neurons from different cell types by over-expression of one TF alone or combined TFs.

TFs	Cell Type	Methods		Major Findings	Ref.
**Nurr1**	Mouse ESCs D3 cell line	In vitro	Overexpress Nurr1	DA neurons markers: Dat, Aadc, Th and Pitx3, Increase the number of DA neurons and the expression of DA markers DA production and release	[81]
	Mouse ESCs R1 cell line	In vitro	Overexpress Nurr1	DA neurons markers: Th, Aadc, Nurr1 and Pitx3 Increase in the proportion of Th^+^ neurons	[82]
		In vivo	Transplantation Nurr1-overexpressed MSCs into PD animals	DA production and release Amelioration of PD motor symptoms in a rodent PD model for 8 weeks Similar electrophysiological characteristics to mesencephalic neurons	
	Mouse ESCs R1 cell line	In vitro	Co-express Nurr1 and GPX-1	DA-like cells: Nestin, Map2 and Tau; Nurr1, DdC, and Th DA release A proportion of immature DA-like cells	[83]
	Rat NPCs derived from cortices (E13.5)	In vitro	Exposure of exogenous Nurr1-expressing NPCs to UCN	Increase the expression of mature DA neurons markers: Dat, Aadc, Th Up-regulate the expression of Nurr1, Foxa2, and Pitx3 Increase Th^+^ cells rather than Nurr1^+^ cells	[85]
	Rat embryo (E16.5)	In vivo	UCN intraperitoneal administration	Increase the differentiation of Nurr1^+^ precursors into Th^+^ DA neurons	
	Primary microglia	In vitro	Exposure of exogenous Nurr1-expressing primary microglia to LPS	Downregulate inflammatory factors (IL-1 and TNFα) Up-regulate neurotrophic factors (BDNF and GDNF)	[86,87]
	Rat mNSCs (E14.5)	In vitro	NNSC + NMG co-culture	DA neurons markers: Th, Pitx3, Dat	[87]
	Rat mNSCs (E12.5–14.5)	In vitro	Overexpress Nurr1 in NSCs	DA neurons markers: Th, Dat	[86]
		In vivo	Transplantation of NNSC + NMG into PD rats	DA neurons markers: Th, Dat, and Pitx3 in the grafts Reduce the number of reactive microglia after transplantation Reverse motor behavior deficits Ensure a long-time significant outcome	
	Rat bone marrow mesenchymal stem cells (MSCs) (6w)	In vivo	Transplantation Nurr1-overexpressed MSCs into PD rats	DA neurons: Nurr1, Th, and Dat Survive and migrate in the brain, Suppress the activation of neuroglial cells and the expression of pro-inflammatory factors Reverse motor behavior deficits	[89]
	Mouse embryonic OBSCs (E13.5)	In vitro	Forced Nurr1 Expression in OBSCs	Mature-like mesencephalic neurons: Th, GIRK2, Vmat2, Dat, calretinin, calbindin DA-GABAergic neurons: Th, GAD, GABA, VGAT DA release A proportion of immature neurons	[90]
**Pitx3**	NPs derived from mouse blastocyst-derived ES cell line J1 (ES-NP)	In vitro	Overexpression of Pitx3 in Shh/Fgf8 pretreated NPs	DA neurons Th, Aadc, Vmat2, and Dat Directly bind to the Th, Ngn2, and Tuj1 gene promoter and induce their transcription.	[91]
	Human ESC line H9	In vivo	Transplantation PITX3-eGFP^+^ cell into PD rats	Not restore functional deficits in PD rats Reduced size of the PITX3-GFP^+^ cell grafts	[92]
		In vivo	Transplantation PITX3-eGFP-cell into PD rats	Restore functional deficits	
	Human teratocarcinoma cell line Ntera2 (NT2)	In vitro	Culture in a growth medium supplemented with knock-out serum and retinoic acid exposed to GDNF	DA neurons markers: Th, Aadc, Dat DA release Accelerate Th expression and induce DA signaling Promote neuroprotection	[93]
		In vivo	Transplantation NT2 cells transduced with Pitx3 into PD rats	Promote neuroprotection Increase in striatal volume. Restore functional deficits	
**Lmx1a**	H9-derived human neural progenitor cell line (hNP1)	In vitro	Forced Lmx1a expression in hNP1	Increase Th^+^ neurons both during NPC and induction stages	[15]
	Human ESC line H9	In vivo	Transplantation Lmx1a-eGFP^+^ VM progenitors from human ESC line into PD rats	Improve the Safety and Predictability Accelerate behavioral recovery Highly enrich for DA neurons Reduce proliferating cell populations Eliminate serotonergic neurons	[92]
	EBCs (embryoid body cells) derived from the R1B5 ESC line	In vivo	Transplantation EBCs transduced with Lmx1a into the intact SNpc	DA neurons Th^+^ cells derived only from EBCs exogenously expressing Lmx1a Additional factors appear to be required to complete differentiation and/or increase the long-term survival of these putative	[94]
		In vivo	Transplantation into the lesioned SNpc	DA neurons The emergence of Th^+^ cells from EBCs Fail to long-term survival	
**Nurr1 + Foxa2**	NPCs from mouse VM and cortex (E10–12)	In vitro	Co-express Nurr1 and Foxa2 in NPCs	Increase Th^+^ cells Synergistic increase of DA genes: Dat, Th, Vmat2, Tuj1, Map2, Pitx3	[48]
	NPCs from mouse VM (E10–12)	In vivo	Co-express Nurr1 and Foxa2 in NPCs	Resistance to toxic stimuli Restoration of PD motor symptoms in a rodent PD model for a long time	[49]
	iNPCs from rat fibroblasts (E13.5)	In vitro	The combined expression of Nurr1 and Foxa2 in iNPCs	Mature/functional DA neuron neurons: Map2, HuC/D, synapsin l, Dat, Vmat2, Th, Tuj1, Pitx3 Action potentials DA release Restore functional deficits The difference in the maturity and function of DA neurons derived from iNPCs in vivo in transplanted brains	[95]
	NPCs from cortices of rat embryos (E14)	In vitro	Forced expression of Nurr1 and Foxa2 using lenti-pUb and retro-pLTR systems	Mature DA neuron generation Mature neurons (Map2, NeuN) Mature DA neurons (Dat, Vmat2) Midbrain-specific DA neurons (Pitx3)	[96]
	Ctx-Ast or VM-Ast	In vitro	Co-culture with VM-NPCs (mouse E10.5 or rat E12) Or co-graft with VM-NPCs	DA release increase Further potentiates the neuroprotective actions Downregulate pro-inflammatory cytokines (IL-1b, IL-6, TNF-α, and iNOS) and myelin-associated proteins (Mbp, Mag, and Mog) Up-regulate secretory neurotrophic (Shh, BDNF, GDNF, and NT3) and anti-inflammatory (TNF-α, TNF-ß) factors Increase antioxidant enzymes	[97]
**Nurr1 + Mash1**	Rat cortical or VM NPCs (E14) and human NPC derived from human ESC line H9	In vitro	Exposure of Nurr1-Mash1-overexpressing NPCs to thyroid hormone derivatives	DA release DA neurons markers: Th, Dat,Nurr1,En1,Lmx1b Protect DA neurons from neurotoxic damage Not affect astrocytes or non-DA neurons	[84]
**Nurr1 + Ngn2**	Mouse embryonic OBSCs E13.5	In vitro	Co-expressing Nurr1 and Ngna2 in OBSCs	Reduction in Th^+^ neural proportion SV2 and Synapsin	[90]
	Astrocytes	In vivo	Viral injection after stab wound injury	NeuN^+^ cells Induced neurons originate from both proliferating and quiescent astrocytes lamina-specific hallmarks Appropriate long-distance axonal projections White matter astrocytes fail to undergo neuronal reprogramming	[98]
**Nurr1 + Pitx3**	iPSCs from Mouse embryonic fibroblasts (MEFs)	In vitro	Transduce iPSCs by Nurr1- and Pitx3-harboring lentiviruses	DA-like cells: Th, Ddc, Dat, Map2 DA release increase	[99]
**Ascl1 + Nurr1**	MEFs	In vitro	Infect MEFs with inducible lentiviruses expressing miR-34b/c cluster in combination with *Ascl1* and *Nurr1*	DA neurons increase Spontaneous electrical activity Up-regulate DA markers: Th, Dat, Vmat2, and Pitx3	[100]
**Brn2, Ascl1, and Myt1L (BAM)**	Mouse or rat fibroblasts (E13.5)	In vitro	Co-express BAM and Bcl-xL in fibroblasts	iNPCs: Tuj1, nestin, Sox2 Features of neural lineage cells. No regional identity and neuronal subtype differentiation potential Mature DA neurons from iNPCs overexpressed Nurr1 and Foxa2	[95]
	Shh overexpressing MS5 stromal (MS5-Shh) cells	In vitro	MS5-Shh cells transduced with BAM and Bcl-xL Co-culture with fibroblasts	DA neurons	[101]
**Ascl1 + Lmx1a + Nurr1 (ALN)**	Primary postnatal mouse astrocytes (strain CD1)	In vitro	Co-expression ALN in Astrocytes	Functional DA Neurons: Tuj1, Pitx3, Lmx1a, En1, aldehyde dehydrogenase, Foxa2, Vmat2, Msx1, and Dat DA release Spontaneous firing of action potentials	[102]
	Mouse (E14.5) and human fibroblasts (IMR90)	In vitro	Co-express ALN in fibroblasts	Functional DA neurons: Th, Vmat2, Dat, ALDH1A1, calbindin, Release DA Spontaneous electrical activity Down-regulation of the fibroblast markers: Twist2, Zeb2, Tgfb1i1, and Chd2 Establishment of DA synaptic terminals	[103]
		In vivo	Transplant fibroblasts transduced ALN into the ventricle of newborn mouse brains.	iDANs markers: Th, Aadc, Vmat2, Dat Maintain excitability and major currents	
	Human Fetal- and Stem Cell-Derived Glial Progenitor Cells	In vitro	Transduced together with short hairpin (sh) RNA against the RE1-silencing transcription factor (REST) complex into cells	Functionally mature iDANs Glial markers down-regulation DA-related genes: Th, Dat, Foxa2, Lmx1a, and Pitx3 Spontaneous firing at resting membrane potential	[104]
	Rat embryonal cortex at E18.5	In vitro	Transduced ALN into Rat embryonal cortex	Mature DA neurons: Th, NeuN, Aadc, Vmat2, Dat Fail to detect secreted DA, DOPAC or HVA DA reprogramming takes place only in GABAergic cortical neurons	[105]
**Pitx3 + Foxa2 + Lmx1a**	H9-derived human neural progenitor cell line (hNP1)	In vitro	Transduced Pitx3, Foxa2, Lmx1a mRNA vectors into hNP1	Th^+^ neurons Midbrain-specific markers: Nurr1, Vmat2	[15]
**Neurod1 + Ascl1 + Lmx1a + miR-218 (NeAL218)**	Human immature astrocytes	In vitro	Reprogram human astrocytes with NeAL218	iDANs: Th^+^/TUBB3^+^ cells Neuronal morphology Lacked membrane properties of excitable DAs	[16]
	Mouse astrocytes	In vivo	Inject NeAL218 lentiviruses into PD mouse	iDANs without tumors or died No GAD1/2, somatostatin (SST), parvalbumin (PVALB), or calretinin (CALB2) expression in iDANs Mature DA markers: Dat, RBFOX3, Nurr1, and PBX1 DA release Rescue spontaneous motor behavior	
**Foxa2 + En1 + Lmx1a + Pitx3**	Human ESCs (H9) or mouse ESCs	In vitro	Coexpress Foxa2, En1, Lmx1a, and Pitx3 in human ESCs (H9) or mouse ESCs	Functional iDANs cells with midbrain characteristics 70% of the Map2^+^ Th^+^ induced neural cells Express Ascl1, Nurr1, Lmx1a, En1, Foxa2, and Pitx3 in Th^+^ induced neural cells DA neurons markers: Th, Vmat2, Dat, Aadc, and Girk2 DA release Electrical properties typical of mesencephalic DA neurons,	[17]
**Sox2 + Nurr1 + Lmx1a + Foxa2**	Striatal Neurons in the Adult Mouse Brain	In vivo	Inject virus including Sox2, Nurr1, Lmx1a and Foxa2 into adult mouse striatal; VPA in drinking water was administered	Mouse striatal neurons are reprogrammed into induced dopaminergic neuron-like cells (iDALs) without a proliferative progenitor stage DA neurons markers: Ddc, Vamt2, Dat, Th without other neuronal subtypes such as ChAT and VGLUT1. iDALs originate from local striatal neurons Electrophysiological properties similar to DA neurons Firing patterns stereotypical to DA neuron Form functional connections with other neurons	[106]
**Ascl1 + Ngn2 + Sox2 + Nurr1 + Pitx3**	IMR90 human fibroblasts	In vitro	Co-express Ascl1, Ngn2, Sox2, Nurr1, Pitx3 in IMR90 human fibroblasts	DA Neurons Markers: Ddc, Vamt2, Dat, Th, En1 Negative for serotonin (a marker for serotogenic neurons) and ChAT (a marker for cholinergic neurons) Lack of cell proliferation DA uptake and production DA neuron-like electrophysiology Relief of PD symptoms	[107]
**Ascl1 + Pitx3 + Nurr1 + Lmx1a + En1 + Foxa2**	Tail tip fibroblasts (TTFs) from adult mice	In vitro	Co-express Ascl1, Pitx3, Nurr1, Lmx1a, En1and Foxa2 in TTFs	DA neuronal marker: Aadc, Vamt2, Dat, Th Mature neuronal makers: Map2 No 5-HT and motor neurons DA release DA neuron-like electrophysiology	[108]
		In vivo	Transplant Pitx3-eGFP^+^ cells isolated from TTFs 12 after transduction with 6 factors into PD models	Neuronal morphology cc DA neuron markers: Th, Aadc Relief of PD symptoms Increase DA production	
**Oct4 + Sox2 + Klf4 + c-Myc**	Human BJ dermal fibroblasts (hDF)	In vitro	spotting culture and quercetin treatment after forced expression Oct4, Sox2, Klf4, c-Myc, miR302s and miR200c in hDF	DA neuronal marker: Th, Dat, Pitx3, Vmat2 DA neuron-like electrophysiology	[109]
		In vivo	Transplate these iPSCs-derived NPCs into PD models	Relief of PD symptoms Form functional connections with other neurons DA neuronal markers: Th, Dat, Vmat2, Nurr1	

Abbreviations: NeuN (Neuronal nuclei); DA (Dopamine); PD (Parkinson’s disease); MAP2 (Microtubule association protein-2); GIRK2 (G-protein–regulated inward-rectifier potassium channel 2); GAD (Glutamate Decarboxylase); GABA (γ-amino butyric acid); VGAT (Vesicular GABA transporter); Tuj1 (Neuronal Class III β-Tubulin); MEFs (Mouse embryonic fibroblasts); iPSCs (Induced pluripotent stem cells); NPs (Neural progenitors); NNSC + NMG (NSCs and microglia both with Nurr1 overexpression); IL-1 (Interleukin-1); TNF-α (Tumor necrosis factor-alpha); LPS (Lipopolysaccharide); GPX-1 (Glutathione peroxidase 1); mNSCs (Mesencephalic neural stem cells); MSCs (Mesenchymal stem cells); NPCs (Neural progenitor cells); UCN (Urocortin); DA neurons (Dopaminergic neurons); iNPCs (induced neural progenitor cells); iDANs (Induced dopaminergic neurons); Dat (DA transporter); Aadc (Amino acid decarboxylase); Th (Tyrosine hydroxylase); Nurr1 (Nuclear receptor-related factor1); Foxa2 (Forkhead box A2); Ascl1 (Aldehyde Dehydrogenase 1 family member A1); Fgf8 (Fibroblast growth factor 8); En1 (Engrailed-1); Pitx3 (Pituitary homeobox 3); PD (Parkinson’s Disease); Neurod1 (Neuronal differentiation); Ngn2 (Neuro-genin 2);Shh (Sonic hedgehog); Vmat2 (Vesicular monoamine transporter 2); BDNF (Brain-derived neurotrophic factor); GDNF (Glial cell line-derived neurotrophic factor); Mash1 (Mammalian achaete-scute homologue-1); iNOS (Inducible nitric oxide synthase); OBSCs (Olfactory bulb stem cells); SV2 (Synaptic vesicle protein 2); Msx1 (Muscle segment homeobox); ALDH1A1 (Recombinant aldehyde dehydrogenase 1 family, member A1); DOPAC (3,4-dihydroxyphenylacetic acid); HVA (Valproic acid); Tubb3 (Tubulin beta 3 class III); Girk2 (G-protein–regulated inward-rectifier potassium channel 2); ChAT (Choline acetyltransferase); 5-HT (5-hydroxy tryptamine); Ctx-Ast (Astrocytes derived from embryonic cortices); VM-Ast (Astrocytes derived from embryonic midbrain); VM (Venteral Mesencephalic).

## Data Availability

Not applicable.

## References

[1] Poewe W., Seppi K., Tanner C.M., Halliday G.M., Brundin P., Volkmann J., Schrag A.-E., Lang A.E. (2017). Parkinson disease. Nat. Rev. Dis. Primers.

[2] Takahashi K., Mizuno K., Sasaki A.T., Wada Y., Tanaka M., Ishii A., Tajima K., Tsuyuguchi N., Watanabe K., Zeki S. (2015). Imaging the passionate stage of romantic love by dopamine dynamics. Front. Hum. Neurosci..

[3] Gebauer L., Kringelbach M.L., Vuust P. (2012). Ever-changing cycles of musical pleasure: The role of dopamine and anticipation. Psychomusicol. Music Mind Brain.

[4] Pascoli V., Terrier J., Hiver A., Lüscher C. (2015). Sufficiency of mesolimbic dopamine neuron stimulation for the progression to addiction. Neuron.

[5] Da Silva J.A., Tecuapetla F., Paixão V., Costa R.M. (2018). Dopamine neuron activity before action initiation gates and invigorates future movements. Nature.

[6] Schultz W. (2007). Multiple dopamine functions at different time courses. Annu. Rev. Neurosci..

[7] Zeng X.S., Geng W.S., Jia J.J., Chen L., Zhang P.P. (2018). Cellular and Molecular Basis of Neurodegeneration in Parkinson Disease. Front. Aging Neurosci..

[8] Schapira A.H.V., Chaudhuri K.R., Jenner P. (2017). Non-motor features of Parkinson disease. Nat. Rev. Neurosci..

[9] Franco R., Reyes-Resina I., Navarro G. (2021). Dopamine in Health and Disease: Much More Than a Neurotransmitter. Biomedicines.

[10] Nemade D., Subramanian T., Shivkumar V. (2021). An Update on Medical and Surgical Treatments of Parkinson’s Disease. Aging Dis..

[11] Le W.-D. (2021). Inauguration of a unique journal *Ageing and Neurodegenerative Diseases*: A new beginning seeking cures for age-related neurodegenerative diseases. Ageing Neurodegener. Dis..

[12] Carmichael K., Sullivan B., Lopez E., Sun L., Cai H. (2021). Diverse midbrain dopaminergic neuron subtypes and implications for complex clinical symptoms of Parkinson’s disease. Ageing Neurodegener. Dis..

[13] Barbuti P.A., Barker R.A., Brundin P., Przedborski S., Papa S.M., Kalia L.V., Mochizuki H. (2021). Recent Advances in the Development of Stem-Cell-Derived Dopaminergic Neuronal Transplant Therapies for Parkinson’s Disease. Mov. Disord..

[14] Zakrzewski W., Dobrzyński M., Szymonowicz M., Rybak Z. (2019). Stem cells: Past, present, and future. Stem Cell Res. Ther..

[15] Azimi S.M., Sheridan S.D., Ghannad-Rezaie M., Eimon P.M., Yanik M.F. (2018). Combinatorial programming of human neuronal progenitors using magnetically-guided stoichiometric mRNA delivery. eLife.

[16] Rivetti di Val Cervo P., Romanov R.A., Spigolon G., Masini D., Martín-Montañez E., Toledo E.M., La Manno G., Feyder M., Pifl C., Ng Y.H. (2017). Induction of functional dopamine neurons from human astrocytes in vitro and mouse astrocytes in a Parkinson’s disease model. Nat. Biotechnol..

[17] Ng Y.H., Chanda S., Janas J.A., Yang N., Kokubu Y., Südhof T.C., Wernig M. (2021). Efficient generation of dopaminergic induced neuronal cells with midbrain characteristics. Stem Cell Rep..

[18] Lambert S.A., Jolma A., Campitelli L.F., Das P.K., Yin Y., Albu M., Chen X., Taipale J., Hughes T.R., Weirauch M.T. (2018). The Human Transcription Factors. Cell.

[19] Canals I., Quist E., Ahlenius H. (2021). Transcription Factor-Based Strategies to Generate Neural Cell Types from Human Pluripotent Stem Cells. Cell Reprogram.

[20] Mesman S., Smidt M.P. (2020). Acquisition of the Midbrain Dopaminergic Neuronal Identity. Int. J. Mol. Sci..

[21] Rodríguez-Traver E., Solís O., Díaz-Guerra E., Ortiz Ó., Vergaño-Vera E., Méndez-Gómez H.R., García-Sanz P., Moratalla R., Vicario-Abejón C. (2016). Role of Nurr1 in the Generation and Differentiation of Dopaminergic Neurons from Stem Cells. Neurotox Res..

[22] Romanos M., Allio G., Roussigné M., Combres L., Escalas N., Soula C., Médevielle F., Steventon B., Trescases A., Bénazéraf B. (2021). Cell-to-cell heterogeneity in Sox2 and Bra expression guides progenitor motility and destiny. eLife.

[23] Kadoya M., Sasai N. (2019). Negative Regulation of mTOR Signaling Restricts Cell Proliferation in the Floor Plate. Front. Neurosci..

[24] Wang M., Ling K.H., Tan J.J., Lu C.B. (2020). Development and Differentiation of Midbrain Dopaminergic Neuron: From Bench to Bedside. Cells.

[25] Bond A.M., Bhalala O.G., Kessler J.A. (2012). The dynamic role of bone morphogenetic proteins in neural stem cell fate and maturation. Dev. Neurobiol..

[26] Arenas E. (2008). Foxa2: The rise and fall of dopamine neurons. Cell Stem Cell.

[27] Tao Y., Zhang S.C. (2016). Neural Subtype Specification from Human Pluripotent Stem Cells. Cell Stem Cell.

[28] Maury Y., Côme J., Piskorowski R.A., Salah-Mohellibi N., Chevaleyre V., Peschanski M., Martinat C., Nedelec S. (2015). Combinatorial analysis of developmental cues efficiently converts human pluripotent stem cells into multiple neuronal subtypes. Nat. Biotechnol..

[29] Allodi I., Hedlund E. (2014). Directed midbrain and spinal cord neurogenesis from pluripotent stem cells to model development and disease in a dish. Front. Neurosci..

[30] Fasano C.A., Chambers S.M., Lee G., Tomishima M.J., Studer L. (2010). Efficient derivation of functional floor plate tissue from human embryonic stem cells. Cell Stem Cell.

[31] Joyner A.L., Liu A., Millet S. (2000). Otx2, Gbx2 and Fgf8 interact to position and maintain a mid-hindbrain organizer. Curr. Opin. Cell Biol..

[32] Puelles E., Annino A., Tuorto F., Usiello A., Acampora D., Czerny T., Brodski C., Ang S.L., Wurst W., Simeone A. (2004). Otx2 regulates the extent, identity and fate of neuronal progenitor domains in the ventral midbrain. Development.

[33] Kim J.Y., Lee J.S., Hwang H.S., Lee D.R., Park C.Y., Jung S.J., You Y.R., Kim D.S., Kim D.W. (2018). Wnt signal activation induces midbrain specification through direct binding of the beta-catenin/TCF4 complex to the EN1 promoter in human pluripotent stem cells. Exp. Mol. Med..

[34] Yang J., Brown A., Ellisor D., Paul E., Hagan N., Zervas M. (2013). Dynamic temporal requirement of Wnt1 in midbrain dopamine neuron development. Development.

[35] Ásgrímsdóttir E.S., Arenas E. (2020). Midbrain Dopaminergic Neuron Development at the Single Cell Level: In vivo and in Stem Cells. Front. Cell Dev. Biol..

[36] Gaggi G., Di Credico A., Izzicupo P., Iannetti G., Di Baldassarre A., Ghinassi B. (2021). Chemical and Biological Molecules Involved in Differentiation, Maturation, and Survival of Dopaminergic Neurons in Health and Parkinson’s Disease: Physiological Aspects and Clinical Implications. Biomedicines.

[37] Blaess S., Ang S.L. (2015). Genetic control of midbrain dopaminergic neuron development. Wiley Interdiscip. Rev. Dev. Biol..

[38] Nakatani T., Kumai M., Mizuhara E., Minaki Y., Ono Y. (2010). Lmx1a and Lmx1b cooperate with Foxa2 to coordinate the specification of dopaminergic neurons and control of floor plate cell differentiation in the developing mesencephalon. Dev. Biol..

[39] Yan C.H., Levesque M., Claxton S., Johnson R.L., Ang S.L. (2011). Lmx1a and lmx1b function cooperatively to regulate proliferation, specification, and differentiation of midbrain dopaminergic progenitors. J. Neurosci..

[40] Deng Q., Andersson E., Hedlund E., Alekseenko Z., Coppola E., Panman L., Millonig J.H., Brunet J.F., Ericson J., Perlmann T. (2011). Specific and integrated roles of Lmx1a, Lmx1b and Phox2a in ventral midbrain development. Development.

[41] Veenvliet J.V., Dos Santos M.T., Kouwenhoven W.M., von Oerthel L., Lim J.L., van der Linden A.J., Koerkamp M.J., Holstege F.C., Smidt M.P. (2013). Specification of dopaminergic subsets involves interplay of En1 and Pitx3. Development.

[42] Lin W., Metzakopian E., Mavromatakis Y.E., Gao N., Balaskas N., Sasaki H., Briscoe J., Whitsett J.A., Goulding M., Kaestner K.H. (2009). Foxa1 and Foxa2 function both upstream of and cooperatively with Lmx1a and Lmx1b in a feedforward loop promoting mesodiencephalic dopaminergic neuron development. Dev. Biol..

[43] Metzakopian E., Bouhali K., Alvarez-Saavedra M., Whitsett J.A., Picketts D.J., Ang S.L. (2015). Genome-wide characterisation of Foxa1 binding sites reveals several mechanisms for regulating neuronal differentiation in midbrain dopamine cells. Development.

[44] Simeone A., Puelles E., Omodei D., Acampora D., Di Giovannantonio L.G., Di Salvio M., Mancuso P., Tomasetti C. (2011). Otx genes in neurogenesis of mesencephalic dopaminergic neurons. Dev. Neurobiol..

[45] Omodei D., Acampora D., Mancuso P., Prakash N., Di Giovannantonio L.G., Wurst W., Simeone A. (2008). Anterior-posterior graded response to Otx2 controls proliferation and differentiation of dopaminergic progenitors in the ventral mesencephalon. Development.

[46] Zetterström R.H., Solomin L., Jansson L., Hoffer B.J., Olson L., Perlmann T. (1997). Dopamine neuron agenesis in Nurr1-deficient mice. Science.

[47] Jankovic J., Chen S., Le W.D. (2005). The role of Nurr1 in the development of dopaminergic neurons and Parkinson’s disease. Prog. Neurobiol..

[48] Yi S.H., He X.B., Rhee Y.H., Park C.H., Takizawa T., Nakashima K., Lee S.H. (2014). Foxa2 acts as a co-activator potentiating expression of the Nurr1-induced DA phenotype via epigenetic regulation. Development.

[49] Oh S.M., Chang M.Y., Song J.J., Rhee Y.H., Joe E.H., Lee H.S., Yi S.H., Lee S.H. (2015). Combined Nurr1 and Foxa2 roles in the therapy of Parkinson’s disease. EMBO Mol. Med..

[50] Smidt M.P., van Schaick H.S., Lanctôt C., Tremblay J.J., Cox J.J., van der Kleij A.A., Wolterink G., Drouin J., Burbach J.P. (1997). A homeodomain gene Ptx3 has highly restricted brain expression in mesencephalic dopaminergic neurons. Proc. Natl. Acad. Sci. USA.

[51] Wang Y., Chen X., Wang Y., Li S., Cai H., Le W. (2021). The essential role of transcription factor Pitx3 in preventing mesodiencephalic dopaminergic neurodegeneration and maintaining neuronal subtype identities during aging. Cell Death Dis..

[52] Maxwell S.L., Ho H.Y., Kuehner E., Zhao S., Li M. (2005). Pitx3 regulates tyrosine hydroxylase expression in the substantia nigra and identifies a subgroup of mesencephalic dopaminergic progenitor neurons during mouse development. Dev. Biol..

[53] Kouwenhoven W.M., von Oerthel L., Smidt M.P. (2017). Pitx3 and En1 determine the size and molecular programming of the dopaminergic neuronal pool. PLoS ONE.

[54] Albéri L., Sgadò P., Simon H.H. (2004). Engrailed genes are cell-autonomously required to prevent apoptosis in mesencephalic dopaminergic neurons. Development.

[55] Blaudin de Thé F.X., Rekaik H., Prochiantz A., Fuchs J., Joshi R.L. (2016). Neuroprotective Transcription Factors in Animal Models of Parkinson Disease. Neural Plast..

[56] Bodea G.O., Spille J.H., Abe P., Andersson A.S., Acker-Palmer A., Stumm R., Kubitscheck U., Blaess S. (2014). Reelin and CXCL12 regulate distinct migratory behaviors during the development of the dopaminergic system. Development.

[57] Brignani S., Raj D.D.A., Schmidt E.R.E., Düdükcü Ö., Adolfs Y., De Ruiter A.A., Rybiczka-Tesulov M., Verhagen M.G., van der Meer C., Broekhoven M.H. (2020). Remotely Produced and Axon-Derived Netrin-1 Instructs GABAergic Neuron Migration and Dopaminergic Substantia Nigra Development. Neuron.

[58] Yang S., Edman L.C., Sánchez-Alcañiz J.A., Fritz N., Bonilla S., Hecht J., Uhlén P., Pleasure S.J., Villaescusa J.C., Marín O. (2013). Cxcl12/Cxcr4 signaling controls the migration and process orientation of A9-A10 dopaminergic neurons. Development.

[59] Vaswani A.R., Weykopf B., Hagemann C., Fried H.U., Brüstle O., Blaess S. (2019). Correct setup of the substantia nigra requires Reelin-mediated fast, laterally-directed migration of dopaminergic neurons. eLife.

[60] Yin M., Liu S., Yin Y., Li S., Li Z., Wu X., Zhang B., Ang S.L., Ding Y., Zhou J. (2009). Ventral mesencephalon-enriched genes that regulate the development of dopaminergic neurons in vivo. J. Neurosci..

[61] Panman L., Papathanou M., Laguna A., Oosterveen T., Volakakis N., Acampora D., Kurtsdotter I., Yoshitake T., Kehr J., Joodmardi E. (2014). Sox6 and Otx2 control the specification of substantia nigra and ventral tegmental area dopamine neurons. Cell Rep..

[62] Simeone A., Di Salvio M., Di Giovannantonio L.G., Acampora D., Omodei D., Tomasetti C. (2011). The role of otx2 in adult mesencephalic-diencephalic dopaminergic neurons. Mol. Neurobiol..

[63] Zheng M., Jiao L., Tang X., Xiang X., Wan X., Yan Y., Li X., Zhang G., Li Y., Jiang B. (2017). Tau haploinsufficiency causes prenatal loss of dopaminergic neurons in the ventral tegmental area and reduction of transcription factor orthodenticle homeobox 2 expression. FASEB J. Off. Publ. Fed. Am. Soc. Exp. Biol..

[64] Hallett P.J., Cooper O., Sadi D., Robertson H., Mendez I., Isacson O. (2014). Long-term health of dopaminergic neuron transplants in Parkinson’s disease patients. Cell Rep..

[65] Brundin P., Pogarell O., Hagell P., Piccini P., Widner H., Schrag A., Kupsch A., Crabb L., Odin P., Gustavii B. (2000). Bilateral caudate and putamen grafts of embryonic mesencephalic tissue treated with lazaroids in Parkinson’s disease. Brain J. Neurol..

[66] Brundin P., Karlsson J., Emgård M., Schierle G.S., Hansson O., Petersén A., Castilho R.F. (2000). Improving the survival of grafted dopaminergic neurons: A review over current approaches. Cell Transplant.

[67] Haas S.J., Beckmann S., Petrov S., Andressen C., Wree A., Schmitt O. (2007). Transplantation of immortalized mesencephalic progenitors (CSM14.1 cells) into the neonatal parkinsonian rat caudate putamen. J. Neurosci. Res..

[68] Jin G., Tan X., Tian M., Qin J., Zhu H., Huang Z., Xu H. (2005). The controlled differentiation of human neural stem cells into TH-immunoreactive (ir) neurons in vitro. Neurosci. Lett..

[69] Saucedo-Cardenas O., Quintana-Hau J.D., Le W.D., Smidt M.P., Cox J.J., De Mayo F., Burbach J.P., Conneely O.M. (1998). Nurr1 is essential for the induction of the dopaminergic phenotype and the survival of ventral mesencephalic late dopaminergic precursor neurons. Proc. Natl. Acad. Sci. USA.

[70] Jakaria M., Haque M.E., Cho D.Y., Azam S., Kim I.S., Choi D.K. (2019). Molecular Insights into NR4A2(Nurr1): An Emerging Target for Neuroprotective Therapy Against Neuroinflammation and Neuronal Cell Death. Mol. Neurobiol..

[71] Wang Q., Song S., Jiang L., Hong J.-S. (2021). Interplay among norepinephrine, NOX2, and neuroinflammation: Key players in Parkinson’s disease and prime targets for therapies. Ageing Neurodegener. Dis..

[72] Dong J., Liu X., Wang Y., Cai H., Le W. (2020). Nurr1(Cd11bcre) conditional knockout mice display inflammatory injury to nigrostriatal dopaminergic neurons. Glia.

[73] Saijo K., Winner B., Carson C.T., Collier J.G., Boyer L., Rosenfeld M.G., Gage F.H., Glass C.K. (2009). A Nurr1/CoREST pathway in microglia and astrocytes protects dopaminergic neurons from inflammation-induced death. Cell.

[74] Chu Y., Kompoliti K., Cochran E.J., Mufson E.J., Kordower J.H. (2002). Age-related decreases in Nurr1 immunoreactivity in the human substantia nigra. J. Comp. Neurol..

[75] Chu Y., Kordower J.H. (2007). Age-associated increases of α-synuclein in monkeys and humans are associated with nigrostriatal dopamine depletion: Is this the target for Parkinson’s disease?. Neurobiol. Dis..

[76] Heng X., Jin G., Zhang X., Yang D., Zhu M., Fu S., Li X., Le W. (2012). Nurr1 regulates Top IIbeta and functions in axon genesis of mesencephalic dopaminergic neurons. Mol. Neurodegener..

[77] Zaim M., Isik S. (2018). DNA topoisomerase IIβ stimulates neurite outgrowth in neural differentiated human mesenchymal stem cells through regulation of Rho-GTPases (RhoA/Rock2 pathway) and Nurr1 expression. Stem Cell Res. Ther..

[78] Kim S.M., Cho S.Y., Kim M.W., Roh S.R., Shin H.S., Suh Y.H., Geum D., Lee M.A. (2020). Genome-Wide Analysis Identifies NURR1-Controlled Network of New Synapse Formation and Cell Cycle Arrest in Human Neural Stem Cells. Mol. Cells.

[79] Kadkhodaei B., Alvarsson A., Schintu N., Ramsköld D., Volakakis N., Joodmardi E., Yoshitake T., Kehr J., Decressac M., Björklund A. (2013). Transcription factor Nurr1 maintains fiber integrity and nuclear-encoded mitochondrial gene expression in dopamine neurons. Proc. Natl. Acad. Sci. USA.

[80] Jodeiri Farshbaf M., Forouzanfar M., Ghaedi K., Kiani-Esfahani A., Peymani M., Shoaraye Nejati A., Izadi T., Karbalaie K., Noorbakhshnia M., Rahgozar S. (2016). Nurr1 and PPARγ protect PC12 cells against MPP(+) toxicity: Involvement of selective genes, anti-inflammatory, ROS generation, and antimitochondrial impairment. Mol. Cell. Biochem..

[81] Chung S., Sonntag K.C., Andersson T., Bjorklund L.M., Park J.J., Kim D.W., Kang U.J., Isacson O., Kim K.S. (2002). Genetic engineering of mouse embryonic stem cells by Nurr1 enhances differentiation and maturation into dopaminergic neurons. Eur. J. Neurosci..

[82] Kim J.H., Auerbach J.M., Rodríguez-Gómez J.A., Velasco I., Gavin D., Lumelsky N., Lee S.H., Nguyen J., Sánchez-Pernaute R., Bankiewicz K. (2002). Dopamine neurons derived from embryonic stem cells function in an animal model of Parkinson’s disease. Nature.

[83] Terraf P., Babaloo H., Kouhsari S.M. (2017). Directed Differentiation of Dopamine-Secreting Cells from Nurr1/GPX1 Expressing Murine Embryonic Stem Cells Cultured on Matrigel-Coated PCL Scaffolds. Mol. Neurobiol..

[84] Lee E.H., Kim S.M., Kim C.H., Pagire S.H., Pagire H.S., Chung H.Y., Ahn J.H., Park C.H. (2019). Dopamine neuron induction and the neuroprotective effects of thyroid hormone derivatives. Sci. Rep..

[85] Huang H.Y., Chiu T.L., Chang H.F., Hsu H.R., Pang C.Y., Liew H.K., Wang M.J. (2015). Epigenetic regulation contributes to urocortin-enhanced midbrain dopaminergic neuron differentiation. Stem Cells.

[86] Qian Y., Chen X.X., Wang W., Li J.J., Wang X.P., Tang Z.W., Xu J.T., Lin H., Yang Z.Y., Li L.Y. (2020). Transplantation of Nurr1-overexpressing neural stem cells and microglia for treating parkinsonian rats. CNS Neurosci. Ther..

[87] Chen X.X., Qian Y., Wang X.P., Tang Z.W., Xu J.T., Lin H., Yang Z.Y., Song X.B., Lu D., Guo J.Z. (2018). Nurr1 promotes neurogenesis of dopaminergic neuron and represses inflammatory factors in the transwell coculture system of neural stem cells and microglia. CNS Neurosci. Ther..

[88] Abumaree M., Al Jumah M., Pace R.A., Kalionis B. (2012). Immunosuppressive properties of mesenchymal stem cells. Stem Cell Rev. Rep..

[89] Wang X., Zhuang W., Fu W., Wang X., Lv E., Li F., Zhou S., Rausch W.D., Wang X. (2018). The lentiviral-mediated Nurr1 genetic engineering mesenchymal stem cells protect dopaminergic neurons in a rat model of Parkinson’s disease. Am. J. Transl. Res..

[90] Vergaño-Vera E., Díaz-Guerra E., Rodríguez-Traver E., Méndez-Gómez H.R., Solís Ó., Pignatelli J., Pickel J., Lee S.H., Moratalla R., Vicario-Abejón C. (2015). Nurr1 blocks the mitogenic effect of FGF-2 and EGF, inducing olfactory bulb neural stem cells to adopt dopaminergic and dopaminergic-GABAergic neuronal phenotypes. Dev. Neurobiol..

[91] Hong S., Chung S., Leung K., Hwang I., Moon J., Kim K.S. (2014). Functional roles of Nurr1, Pitx3, and Lmx1a in neurogenesis and phenotype specification of dopamine neurons during in vitro differentiation of embryonic stem cells. Stem Cells Dev..

[92] de Luzy I.R., Niclis J.C., Gantner C.W., Kauhausen J.A., Hunt C.P.J., Ermine C., Pouton C.W., Thompson L.H., Parish C.L. (2019). Isolation of LMX1a Ventral Midbrain Progenitors Improves the Safety and Predictability of Human Pluripotent Stem Cell-Derived Neural Transplants in Parkinsonian Disease. J. Neurosci..

[93] Eskandarian Boroujeni M., Aliaghaei A., Maghsoudi N., Gardaneh M. (2020). Complementation of dopaminergic signaling by Pitx3-GDNF synergy induces dopamine secretion by multipotent Ntera2 cells. J. Cell. Biochem..

[94] Collazo-Navarrete O., Hernández-García D., Guerrero-Flores G., Drucker-Colín R., Guerra-Crespo M., Covarrubias L. (2019). The Substantia Nigra Is Permissive and Gains Inductive Signals When Lesioned for Dopaminergic Differentiation of Embryonic Stem Cells. Stem Cells Dev..

[95] Lim M.S., Chang M.Y., Kim S.M., Yi S.H., Suh-Kim H., Jung S.J., Kim M.J., Kim J.H., Lee Y.S., Lee S.Y. (2015). Generation of Dopamine Neurons from Rodent Fibroblasts through the Expandable Neural Precursor Cell Stage. J. Biol. Chem..

[96] Kim T., Song J.J., Puspita L., Valiulahi P., Shim J.W., Lee S.H. (2017). In Vitro generation of mature midbrain-type dopamine neurons by adjusting exogenous Nurr1 and Foxa2 expressions to their physiologic patterns. Exp. Mol. Med..

[97] Song J.J., Oh S.M., Kwon O.C., Wulansari N., Lee H.S., Chang M.Y., Lee E., Sun W., Lee S.E., Chang S. (2018). Cografting astrocytes improves cell therapeutic outcomes in a Parkinson’s disease model. J. Clin. Investig..

[98] Mattugini N., Bocchi R., Scheuss V., Russo G.L., Torper O., Lao C.L., Götz M. (2019). Inducing Different Neuronal Subtypes from Astrocytes in the Injured Mouse Cerebral Cortex. Neuron.

[99] Salemi S., Baktash P., Rajaei B., Noori M., Amini H., Shamsara M., Massumi M. (2016). Efficient generation of dopaminergic-like neurons by overexpression of Nurr1 and Pitx3 in mouse induced Pluripotent Stem Cells. Neurosci. Lett..

[100] De Gregorio R., Pulcrano S., De Sanctis C., Volpicelli F., Guatteo E., von Oerthel L., Latagliata E.C., Esposito R., Piscitelli R.M., Perrone-Capano C. (2018). miR-34b/c Regulates Wnt1 and Enhances Mesencephalic Dopaminergic Neuron Differentiation. Stem Cell Rep..

[101] Lim M.S., Kim S.M., Lee E.H., Park C.H. (2016). Efficient induction of neural precursor cells from fibroblasts using stromal cell-derived inducing activity. Tissue Eng. Regen Med..

[102] Addis R.C., Hsu F.C., Wright R.L., Dichter M.A., Coulter D.A., Gearhart J.D. (2011). Efficient conversion of astrocytes to functional midbrain dopaminergic neurons using a single polycistronic vector. PLoS ONE.

[103] Caiazzo M., Dell’Anno M.T., Dvoretskova E., Lazarevic D., Taverna S., Leo D., Sotnikova T.D., Menegon A., Roncaglia P., Colciago G. (2011). Direct generation of functional dopaminergic neurons from mouse and human fibroblasts. Nature.

[104] Nolbrant S., Giacomoni J., Hoban D.B., Bruzelius A., Birtele M., Chandler-Militello D., Pereira M., Ottosson D.R., Goldman S.A., Parmar M. (2020). Direct Reprogramming of Human Fetal- and Stem Cell-Derived Glial Progenitor Cells into Midbrain Dopaminergic Neurons. Stem Cell Rep..

[105] Raina A., Mahajani S., Bähr M., Kügler S. (2020). Neuronal Trans-differentiation by Transcription Factors Ascl1 and Nurr1: Induction of a Dopaminergic Neurotransmitter Phenotype in Cortical GABAergic Neurons. Mol. Neurobiol..

[106] Niu W., Zang T., Wang L.L., Zou Y., Zhang C.L. (2018). Phenotypic Reprogramming of Striatal Neurons into Dopaminergic Neuron-like Cells in the Adult Mouse Brain. Stem Cell Rep..

[107] Liu X., Li F., Stubblefield E.A., Blanchard B., Richards T.L., Larson G.A., He Y., Huang Q., Tan A.C., Zhang D. (2012). Direct reprogramming of human fibroblasts into dopaminergic neuron-like cells. Cell Res..

[108] Kim J., Su S.C., Wang H., Cheng A.W., Cassady J.P., Lodato M.A., Lengner C.J., Chung C.Y., Dawlaty M.M., Tsai L.H. (2011). Functional integration of dopaminergic neurons directly converted from mouse fibroblasts. Cell Stem Cell.

[109] Song B., Cha Y., Ko S., Jeon J., Lee N., Seo H., Park K.J., Lee I.H., Lopes C., Feitosa M. (2020). Human autologous iPSC-derived dopaminergic progenitors restore motor function in Parkinson’s disease models. J. Clin. Investig..

[110] Bäckström D., Domellöf M.E., Granåsen G., Linder J., Mayans S., Elgh E., Mo S.J., Forsgren L. (2017). PITX3 genotype and risk of dementia in Parkinson’s disease: A population-based study. J. Neurol. Sci..

[111] Tiklová K., Björklund Å.K., Lahti L., Fiorenzano A., Nolbrant S., Gillberg L., Volakakis N., Yokota C., Hilscher M.M., Hauling T. (2019). Single-cell RNA sequencing reveals midbrain dopamine neuron diversity emerging during mouse brain development. Nat. Commun..

[112] Lei Z., Jiang Y., Li T., Zhu J., Zeng S. (2011). Signaling of glial cell line-derived neurotrophic factor and its receptor GFRα1 induce Nurr1 and Pitx3 to promote survival of grafted midbrain-derived neural stem cells in a rat model of Parkinson disease. J. Neuropathol. Exp. Neurol..

[113] D’ANGLEMONT de Tassigny X., Pascual A., López-Barneo J. (2015). GDNF-based therapies, GDNF-producing interneurons, and trophic support of the dopaminergic nigrostriatal pathway. Implications for Parkinson’s disease. Front. Neuroanat..

[114] Peng C., Fan S., Li X., Fan X., Ming M., Sun Z., Le W. (2007). Overexpression of pitx3 upregulates expression of BDNF and GDNF in SH-SY5Y cells and primary ventral mesencephalic cultures. FEBS Lett..

[115] Yang D., Peng C., Li X., Fan X., Li L., Ming M., Chen S., Le W. (2008). Pitx3-transfected astrocytes secrete brain-derived neurotrophic factor and glial cell line-derived neurotrophic factor and protect dopamine neurons in mesencephalon cultures. J. Neurosci. Res..

[116] Failli V., Bachy I., Rétaux S. (2002). Expression of the LIM-homeodomain gene Lmx1a (dreher) during development of the mouse nervous system. Mech. Dev..

[117] Doucet-Beaupré H., Gilbert C., Profes M.S., Chabrat A., Pacelli C., Giguère N., Rioux V., Charest J., Deng Q., Laguna A. (2016). Lmx1a and Lmx1b regulate mitochondrial functions and survival of adult midbrain dopaminergic neurons. Proc. Natl. Acad. Sci. USA.

[118] Salesse C., Charest J., Doucet-Beaupré H., Castonguay A.M., Labrecque S., De Koninck P., Lévesque M. (2020). Opposite Control of Excitatory and Inhibitory Synapse Formation by Slitrk2 and Slitrk5 on Dopamine Neurons Modulates Hyperactivity Behavior. Cell Rep..

[119] Davis C.A., Joyner A.L. (1988). Expression patterns of the homeo box-containing genes En-1 and En-2 and the proto-oncogene int-1 diverge during mouse development. Genes Dev..

[120] Marei H.E., Althani A., Afifi N., Michetti F., Pescatori M., Pallini R., Casalbore P., Cenciarelli C., Schwartz P., Ahmed A.E. (2011). Gene expression profiling of embryonic human neural stem cells and dopaminergic neurons from adult human substantia nigra. PLoS ONE.

[121] Kim T.W., Piao J., Koo S.Y., Kriks S., Chung S.Y., Betel D., Socci N.D., Choi S.J., Zabierowski S., Dubose B.N. (2021). Biphasic Activation of WNT Signaling Facilitates the Derivation of Midbrain Dopamine Neurons from hESCs for Translational Use. Cell Stem Cell.

[122] Kouwenhoven W.M., Veenvliet J.V., van Hooft J.A., van der Heide L.P., Smidt M.P. (2016). Engrailed 1 shapes the dopaminergic and serotonergic landscape through proper isthmic organizer maintenance and function. Biol. Open.

[123] Prochiantz A., Fuchs J., Di Nardo A.A. (2014). Postnatal signalling with homeoprotein transcription factors. Philos. Trans. R. Soc. Lond. Ser. B Biol. Sci..

[124] Rekaik H., Blaudin de Thé F.X., Fuchs J., Massiani-Beaudoin O., Prochiantz A., Joshi R.L. (2015). Engrailed Homeoprotein Protects Mesencephalic Dopaminergic Neurons from Oxidative Stress. Cell Rep..

[125] Nordströma U., Beauvais G., Ghosh A., Pulikkaparambil Sasidharan B.C., Lundblad M., Fuchs J., Joshi R.L., Lipton J.W., Roholt A., Medicetty S. (2015). Progressive nigrostriatal terminal dysfunction and degeneration in the engrailed1 heterozygous mouse model of Parkinson’s disease. Neurobiol. Dis..

[126] Stott S.R., Metzakopian E., Lin W., Kaestner K.H., Hen R., Ang S.L. (2013). Foxa1 and foxa2 are required for the maintenance of dopaminergic properties in ventral midbrain neurons at late embryonic stages. J. Neurosci..

[127] Dong D., Meng L., Yu Q., Tan G., Ding M., Tan Y. (2012). Stable expression of FoxA1 promotes pluripotent P19 embryonal carcinoma cells to be neural stem-like cells. Gene Expr..

[128] Pristerà A., Lin W., Kaufmann A.K., Brimblecombe K.R., Threlfell S., Dodson P.D., Magill P.J., Fernandes C., Cragg S.J., Ang S.L. (2015). Transcription factors FOXA1 and FOXA2 maintain dopaminergic neuronal properties and control feeding behavior in adult mice. Proc. Natl. Acad. Sci. USA.

[129] Domanskyi A., Alter H., Vogt M.A., Gass P., Vinnikov I.A. (2014). Transcription factors Foxa1 and Foxa2 are required for adult dopamine neurons maintenance. Front. Cell. Neurosci..

[130] Mavromatakis Y.E., Lin W., Metzakopian E., Ferri A.L., Yan C.H., Sasaki H., Whisett J., Ang S.L. (2011). Foxa1 and Foxa2 positively and negatively regulate Shh signalling to specify ventral midbrain progenitor identity. Mech. Dev..

[131] Torper O., Ottosson D.R., Pereira M., Lau S., Cardoso T., Grealish S., Parmar M. (2015). In Vivo Reprogramming of Striatal NG2 Glia into Functional Neurons that Integrate into Local Host Circuitry. Cell Rep..

[132] Pereira M., Birtele M., Shrigley S., Benitez J.A., Hedlund E., Parmar M., Ottosson D.R. (2017). Direct Reprogramming of Resident NG2 Glia into Neurons with Properties of Fast-Spiking Parvalbumin-Containing Interneurons. Stem Cell Rep..

[133] Tsai R.Y. (2018). Creating a graft-friendly environment for stem cells in diseased brains. J. Clin. Investig..

[134] He M., Zhang H., Li Y., Tian C., Tang B., Huang Y., Zheng J. (2019). Direct and selective lineage conversion of human fibroblasts to dopaminergic precursors. Neurosci. Lett..

[135] Schweitzer J.S., Song B., Herrington T.M., Park T.Y., Lee N., Ko S., Jeon J., Cha Y., Kim K., Li Q. (2020). Personalized iPSC-Derived Dopamine Progenitor Cells for Parkinson’s Disease. N. Engl. J. Med..

[136] Doi D., Magotani H., Kikuchi T., Ikeda M., Hiramatsu S., Yoshida K., Amano N., Nomura M., Umekage M., Morizane A. (2020). Pre-clinical study of induced pluripotent stem cell-derived dopaminergic progenitor cells for Parkinson’s disease. Nat. Commun..

[137] Doi D., Samata B., Katsukawa M., Kikuchi T., Morizane A., Ono Y., Sekiguchi K., Nakagawa M., Parmar M., Takahashi J. (2014). Isolation of human induced pluripotent stem cell-derived dopaminergic progenitors by cell sorting for successful transplantation. Stem Cell Rep..

[138] Takahashi J. (2020). iPS cell-based therapy for Parkinson’s disease: A Kyoto trial. Regen. Ther..

[139] Takahashi J. (2020). Preclinical evaluation of patient-derived cells shows promise for Parkinson’s disease. J. Clin. Investig..

[140] Park C.H., Kang J.S., Kim J.S., Chung S., Koh J.Y., Yoon E.H., Jo A.Y., Chang M.Y., Koh H.C., Hwang S. (2006). Differential actions of the proneural genes encoding Mash1 and neurogenins in Nurr1-induced dopamine neuron differentiation. J. Cell Sci..

[141] Andersson E.K., Irvin D.K., Ahlsiö J., Parmar M. (2007). Ngn2 and Nurr1 act in synergy to induce midbrain dopaminergic neurons from expanded neural stem and progenitor cells. Exp. Cell Res..

[142] Xia N., Zhang P., Fang F., Wang Z., Rothstein M., Angulo B., Chiang R., Taylor J., Reijo Pera R.A. (2016). Transcriptional comparison of human induced and primary midbrain dopaminergic neurons. Sci. Rep..

